# *Crithidia fasciculata* Shows Non-Pathogenic Behavior in Leishmania Co-Infection Related to Temperature Stress, In Vitro and In Vivo Infections, and Amphotericin B Susceptibility

**DOI:** 10.3390/microorganisms13102335

**Published:** 2025-10-10

**Authors:** Julia Fernandes Barbosa dos Santos, Carolina Boucinha Martins, Valter Viana Andrade-Neto, Thais Lemos-Silva, Rosiane Freire dos Santos, Silvia Amaral Gonçalves da-Silva, Yara Maria Traub-Csekö, Rubem Figueiredo Sadok Menna-Barreto, Eduardo Caio Torres-Santos, Claudia Masini d’Avila, Vitor Ennes-Vidal

**Affiliations:** 1 Laboratório de Doenças Parasitárias, Fundação Oswaldo Cruz, Instituto Oswaldo Cruz, Avenida Brasil 4365, CEP 21040-900, Manguinhos, Rio de Janeiro 21040-360, RJ, Brazil; juferbs@gmail.com (J.F.B.d.S.); masinidavila@gmail.com (C.M.d.); 2 Laboratório de Bioquímica de Tripanosomatídeos, Fundação Oswaldo Cruz, Instituto Oswaldo Cruz, Avenida Brasil 4365, CEP 21040-900, Manguinhos, Rio de Janeiro 21040-360, RJ, Brazil; valterfarma@yahoo.com.br (V.V.A.-N.); ects@ioc.fiocruz.br (E.C.T.-S.); 3 Laboratório de Mosquitos Transmissores de Hematozoários, Fundação Oswaldo Cruz, Instituto Oswaldo Cruz, Avenida Brasil 4365, CEP 21040-900, Manguinhos, Rio de Janeiro 21040-360, RJ, Brazil; lathema@ioc.fiocruz.br; 4 Laboratório de Biologia Molecular de Parasitos e Vetores, Fundação Oswaldo Cruz, Instituto Oswaldo Cruz, Avenida Brasil 4365, CEP 21040-900, Manguinhos, Rio de Janeiro 21040-360, RJ, Brazil; labmpv@ioc.fiocruz.br (T.L.-S.); ytraub@ioc.fiocruz.br (Y.M.T.-C.); 5 Laboratório de Imunofarmacologia Parasitária, Universidade do Estado do Rio de Janeiro, Av. Prof. Manoel de Abreu, 444, CEP 20550-170, Maracanã, Rio de Janeiro 20550-170, RJ, Brazil; rosiannefreire@gmail.com (R.F.d.S.); silvasag@gmail.com (S.A.G.d.-S.); 6 Laboratório de Biologia Celular, Fundação Oswaldo Cruz, Instituto Oswaldo Cruz, Avenida Brasil 4365, CEP 21040-900, Manguinhos, Rio de Janeiro 21040-360, RJ, Brazil; lbc@ioc.fiocruz.br

**Keywords:** *Leishmania*, co-infection, macrophage, sandfly, amphotericin B

## Abstract

There is increasing evidence on the occurrence of *Crithidia* spp. in patients presenting either cutaneous or visceral leishmaniasis, solely or associated with *Leishmania*. We analyzed growth, morphology, and temperature tolerance of two *C. fasciculata* strains, the reference strain COLPROT048 and patient isolate COLPROT606. We also evaluated their co-cultivation with *L. braziliensis*, macrophage infectivity, and infections in hamsters, BALB/c mice, and sandflies. In culture, both *Crithidia* strains survived at 32 °C for 96 h, showing major morphological alterations and decreased mitochondrial membrane potential, with ΔΨm reducing to 52% in COLPROT606. At 34 °C, the patient isolate showed an 80% reduction in cell number. Mixed cultivation of *Crithidia-Leishmania* led to recovery of only *Crithidia*. In macrophages, *C. fasciculata* alone was virtually eliminated, and in co-infection only *Leishmania* was detected. No *Crithidia* lesion or RNA were found in infected mice or hamsters, while *L. braziliensis* reached 1145–1625 parasites/mg of tissue. In sandflies, *C. fasciculata* successfully established infection for up to 7 days, both alone and in coinfections. Amphotericin B IC_50_ values at 72 h were 4- to 5-fold higher in *C. fasciculata* strains compared to *L. braziliensis*. Our results indicate that both *C. fasciculata* strains are unable to reproduce the pathogenic effect in vitro and in vivo models.

## 1. Introduction

Trypanosomatid protozoa constitute a diverse group that includes the etiological agents of several neglected tropical diseases (NTDs) [1]. These parasites are transmitted by a variety of arthropod vectors and cause a wide spectrum of diseases associated with substantial morbidity and mortality [2]. Conventionally, trypanosomatid species are classified into dixenous parasites, which complete their life cycle, alternating between invertebrate and vertebrate or plant hosts; and monoxenous trypanosomatids, assumed to be restricted to invertebrate hosts, mainly insects. While dixenous species have received more attention due to their considerable medical, veterinary, and economic relevance [3], typically nonpathogenic monoxenous trypanosomatids have gained increasing interest due to growing reports of their occurrence in mammalian hosts, including humans [4,5,6,7,8,9,10,11,12,13,14,15,16].

Most of the recent reports of monoxenous infections in mammals have emerged from the atypical nature of some cases, prompting further investigations into the pathogenic species involved. In most instances, these infections occur in co-infection with HIV or other trypanosomatids, such as *Leishmania* [7]. These findings disclose ongoing controversies surrounding their taxonomy and potential pathogenicity in vertebrate hosts. Nevertheless, the impact of monoxenous trypanosomatid in the pathogenesis of leishmaniasis remains poorly understood, raising critical questions about how mixed infections affect disease severity, diagnosis, and treatment.

In this sense, our research group received an isolate from a cutaneous lesion of an immunocompetent man in Cusco, Peru [4]. The parasite was initially identified as *Leishmania braziliensis* based on clinical presentation and geographic distribution, but further studies revealed atypical features, such as non-fastidious cultivation. However, more accurate genomic analysis employing the molecular markers gGAPDH and V7V8 ribosomal RNA region identified the parasite as *Crithidia fasciculata*, which was subsequently deposited in the Protozoa Collection in the Oswaldo Cruz Foundation (COLPROT-Fiocruz) [14].

The isolation of *C. fasciculata* from a human lesion led us to hypothesize that the monoxenous parasite may persist in mammalian hosts and modulate the outcomes of co-infection with *L. braziliensis*. To elucidate this issue, a comprehensive analysis of the growth dynamics and infectivity of *C. fasciculata* isolated from a human patient were performed. The parasite’s behavior in different culture media was assessed, as well as its in vitro and in vivo infectivity against mouse and hamster models. Finally, we examined the potential effect of the co-infection of *C. fasciculata* and *L. braziliensis* on the colonization of the sandfly vector *Lutzomyia longipalpis*, thereby contributing to a better understanding of its potential as a vector-borne pathogen. This study provides valuable information into the ecological and pathological roles of monoxenous trypanosomatids in human infections and advances our knowledge of co-infection mechanisms and the pathogenic potential of *C. fasciculata*.

## 2. Materials and Methods

### 2.1. Parasite Cultivation

The following strains from our Protozoa Collection (COLPROT—Fiocruz) were used: (i) the type strain of *C. fasciculata* (COLPROT048), isolated from *Anopheles quadrimaculatus* in 1926; (ii) *C. fasciculata* (COLPROT606), isolated from a human patient in 1994; and (iii) *L. braziliensis* (ThorMCAN/BR/97/P142) obtained from LBTq/IOC and maintained through passages in BALB/c mice. *C. fasciculata* strains were initially cultivated in NNN’/LIT medium with 10% inactivated FBS, while *L. braziliensis* was maintained in Schneider’s medium (Sigma-Aldrich, St. Louis, MO, USA) with 20% inactivated FBS and 2% sterile human filtered urine at 27 °C. *C. fasciculata* strains were subsequently maintained in Schneider’s medium with 10% inactivated FBS (Gibco) at 27 °C. Passages were performed twice a week until the fifth passage.

### 2.2. Growth Curves

Growth curves were established by inoculating *C. fasciculata* (COLPROT606 and COLPROT048) and *L. braziliensis* (MCAN/BR/97/P142) cultures, starting with 1.0 × 10^6^ parasites/mL in LIT and Schneider’s medium supplemented with 10% and 20% inactivated FBS, respectively. Parasites were maintained in triplicate and monitored every 24 h during five days. Parasite viability was assessed using a Neubauer chamber and Trypan blue (Sigma-Aldrich) exclusion. The effect of temperature on COLPROT606 was evaluated at 27 °C, 32 °C and 34 °C. Replicates were passed during the logarithmic growth phase [17].

Alternatively, to evaluate mixed cultures, *L. braziliensis* and COLPROT606 were co-cultured in Schneider’s medium with 20% inactivated FBS and 2% sterile male human urine at 27 °C in the following ratios: *L. braziliensis* (control), COLPROT606 (control), and three mixed ratios (1:1, 2:1, 1:2). In all mixed cultures, the total number of parasites was equivalent to the corresponding single-species control, with the number of each species adjusted according to the ratio, e.g., 1 × 10^6^ each in 1:1; 1.33 × 10^6^ of one species and 0.67 × 10^6^ of the other in 2:1 or 1:2, totaling 2 × 10^6^ parasites. Cultures were maintained in 5 mL medium, monitored every 3 days for contamination and growth, and 300 µL culture was transferred into fresh medium. After five passages, the cultures were washed three times with sterile PBS, centrifuged, and stored in TRIzol^®^ (Invitrogen, Waltham, MA, USA) at −80 °C for RNA extraction and qPCR analysis [18].

### 2.3. Morphological Analysis

For cell morphology analysis, *C. fasciculata* COLPROT606 and COLPROT048 were stained with Giemsa (Sigma-Aldrich) and observed under a Zeiss AxioObserver M1 optical microscope with a 100 × objective. Smears were prepared from *log* phase samples (5.0 × 10^6^ parasites/mL), fixed in 100% methanol for 10 min, air-dried, treated with 5N HCl for 10 min, washed, and stained with Giemsa for 1 h.

### 2.4. Mitochondrial Membrane Potential (ΔΨm) Assay

To evaluate the impact of temperature on *C. fasciculata* strains, the mitochondrial membrane potential (ΔΨm) of parasites incubated at 32 °C was evaluated by flow cytometry. The parasites were incubated with 50 nM tetramethylrhodamine (TMRE) (Molecular Probes) for 15 min at 28 °C, using 50 μM carbonyl cyanide m-chlorophenyl hydrazone (CCCP) (Sigma, St. Louis, MO, USA) as a control for ΔΨm dissipation. Variations in TMRE fluorescence were quantified using an index of variation (IV) following the equation IV = (ME − MC)/MC, where ME corresponds to the median fluorescence of parasites under experimental conditions of 32 °C and MC corresponds to the median fluorescence of parasites under control conditions at 27 °C. Negative IV values indicate depolarization of the mitochondrial membrane. Fluorescence values of CCCP-treated parasites were subtracted from both ME and MC values [19]. In parallel, the parasites were labeled with 0.1 µM TO-PRO3 iodide (Invitrogen) for 30 min to evaluate the plasma membrane integrity. Parasites incubated in 0.01% Triton X-100 (Sigma) were used as positive control. The parasites (10,000) were kept on ice until the acquisition on Beckman Coulter’s CyAn Flow Cytometer, and finally analyzed in the CytExpert 2.5 software.

### 2.5. Murine Peritoneal Macrophages In Vitro Infection

Peritoneal macrophages were extracted from BALB/c mice and adhered to coverslips (3 × 10^5^ cells) with RPMI 1640 medium (Gibco, Waltham, MA, USA) in a 4% CO_2_ atmosphere at 37 °C for 24 h, and were then washed thrice with PBS. Subsequently, macrophages (1.0 × 10^6^ cells/well) were infected with *L. braziliensis* (MCAN/BR/97/P142 strain) and both *C. fasciculata* strains (COLPROT048 and COLPROT606) at a 5 parasites per macrophage ratio. For co-infections, a 1:1 ratio was employed, yielding 2.5 parasites of each species per macrophage, thereby maintaining the total ratio of 5 parasites per macrophage used in monoinfections. Cells were cultured in RPMI medium supplemented with 10% FBS, 1% glutamine, and 1% pyruvate for 24, 48, 72, and 96 h at 35 °C with 5% CO_2_. After incubation, slides were stained with Panotic Fast (Laborclin, Pinhais, Brazil), and the infection index was determined by counting under an optical microscope using the formula: (% infected macrophages × amastigotes count)/total macrophages [20].

### 2.6. Amphotericin B Sensitivity Assays

For Amphotericin B assays, 4 × 10^6^ parasites/mL were used in 96-well plates. The drug was tested in a serial dilution range starting at 1 μM. After 72 h of incubation at 26 °C, cell viability was assessed by fluorometry using 50 µM Alamar Blue^®^resazurin. Readings were taken with a SpectraMax GEMINI XPS (Molecular Devices, San Jose, CA, USA), with an excitation of 560 nm to an emission detection at 590 nm. All assays were performed in triplicate. The results were plotted as the 50% inhibitory concentration (IC_50_/72 h) for parasite growth, using a nonlinear regression analysis on a semi-logarithmic scale obtained from GraphPad Prism 5.0 software [21].

### 2.7. In Vivo Experimental Infection Models

In vivo experiments were conducted in two models. Female BALB/c mice (4–8 weeks old, *n* = per group) from ICTB Fiocruz, housed at the Oswaldo Cruz Institute (Animal Ethics Committee approval: L002/2022), were infected in the right ear with 2.0 × 10^6^ parasites in the following groups: Group I—*L. braziliensis* (control), Group II –*C. fasciculata* COLPROT048, Group III—*C. fasciculata* COLPROT606, Group IV—*L. braziliensis* + COLPROT 048 (1:1 mixture, 1.0 × 10^6^ of each), and Group V—*L. braziliensis* + COLPROT606 (1:1 mixture, 1.0 × 10^6^ of each). Lesion size was monitored twice weekly for 35 days with a dial caliper (Mitutoyo, Kawasaki-shi, Japan). The animals had the lesion size measured for 78 days post-infection, and were euthanized at the end of the experiment. The infected paw and popliteal lymph nodes were removed and macerated for analysis by limiting dilution (LDA) and qPCR absolute quantification. Parasitic load was evaluated by limiting dilution and RT-qPCR. The second in vivo model was Golden hamsters (6–8 weeks old) from the UERJ animal facility (Animal Ethics Committee approval: 023/2022), which were used in similar experiments, i.e., infected in the right paw with 1.0 × 10^6^ parasites and monitored for lesion size. After euthanasia, the infected paw and popliteal lymph nodes were removed for analysis [21].

For the immunosuppression assays, BALB/c mice (*n* = 10 per group) were treated with cyclophosphamide (150 mg/kg) intraperitoneally, and subsequently infected with 1 × 10^7^ *C. fasciculata* COLPROT048 or COLPROT606. Lesions were monitored weekly, and parasitic load was assessed by limiting dilution.

### 2.8. RT-qPCR Quantification

Parasitic load was quantified using reverse transcriptase quantitative PCR (RT-qPCR). Primers and probes (IDT Inc., Newark, NJ, USA) were designed based on conserved regions of the SSU rRNA gene sequences of *L. braziliensis* and *C. fasciculata* using MUSCLE and Primer Express software v3.0.1. RNA was extracted with TRIzol and RNeasy Mini Kit (Qiagen, Hilden, Germany), treated with DNase (Sigma), and converted to cDNA using the SuperScript IV VILO kit (Invitrogen). The absence of genomic DNA amplification was confirmed by conventional PCR with actin primers in the no-reverse transcriptase control provided by the kit [17]. qPCR was performed on an ABI Prism 7500 Fast system, with the primers and probes described in Table 1. Standard curves were generated from serial dilutions of cDNA (10^7^ to 10^2^ parasites equivalents) to quantify parasitemia and assess primer efficiency. All the reactions were performed in triplicate.

### 2.9. Sandflies Experimental Infection

Infections of sandflies were performed using 5–8-day-old female *L. longipalpis* by artificially feeding on mice blood containing 5.0 × 10^6^ parasites/mL of *L. braziliensis* (MCAN/BR/97/P142), *C. fasciculata* (COLPROT606), or a 1:1 mixture of both parasites (2.5 × 10^6^ of each species). Pools of 10 sandflies from 3- and 7 days post-infection had their RNA extracted and cDNA synthesized by the protocols described previously. The parasite load was evaluated by qPCR using SYBR Green (Invitrogen) and employing the same SSU rRNA primers for the trypanosomatids’ detection and *L. longipalpis* ribosomal protein (RP49) as a normalization control [22]. The parasite load was represented by the number of quantified parasites (*L. braziliensis* or *C. fasciculata*) × 10^4^ per the number of sandflies in the pools (trypanosomatid × 10^4^/sandfly pool).

### 2.10. Statistical Analysis

All data were analyzed using GraphPad Prism 5.0 software (San Diego, CA, USA). For growth curves, viability assays, and drug sensitivity tests, normally distributed data with equal variances were compared using two-way ANOVA followed by Bonferroni’s multiple-comparison post hoc test. For macrophage infection indices and parasite load in animal tissues, comparisons between two groups were performed using Student’s *t*-test when data were normally distributed, or the Mann–Whitney U test otherwise. For sand fly infection and colonization experiments, parasite load was analyzed by two-way ANOVA with Bonferroni’s correction. Values of *p* < 0.05 were considered statistically significant.

## 3. Results

### 3.1. Growth Kinetics of C. fasciculata COLPROT60

**Figure 1:**
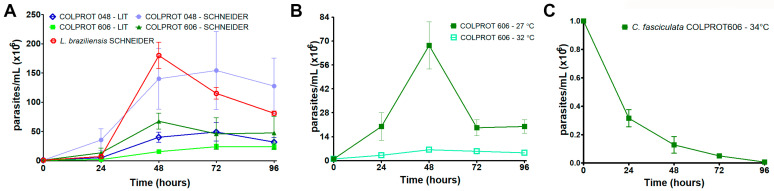
Cell growth kinetics of *L. braziliensis* and *C. fasciculata* COLPROT048 and COLPROT606. (**A**) The *L. braziliensis* and *C. fasciculata* growth pattern at 27 °C in LIT and Schneider‘s medium supplemented with 10% FBS and 20% FBS, respectively; (**B**) *C. fasciculata* COLPROT606 growth at 27 °C and 32 °C in Schneider medium supplemented with 20% FBS. (**C**) *C. fasciculata* COLPROT606 kinetics of growth at 34 °C in Schneider medium supplemented with 20% SFB. An initial inoculum of 1.0 × 10^6^ parasites/mL in the logarithmic phase was used to start the growth curves. Cells were quantified using a Neubauer chamber every 24 h over five days.

The presence of monoxenous co-infections with *Leishmania* should imply several adaptations to the environment of the human body, such as temperature. Pathogenic *Leishmania* species exhibit intracellular amastigotes in mammalian cells and extracellular promastigotes in the vector, while *Crithidia* species typically display the choanomastigote form, but promastigotes and intermediate forms may appear in cultures. To investigate the growth kinetic of the isolated *C. fasciculata* COLPROT606, parasites were cultured in LIT medium with 10% FBS and Schneider’s medium with 20% FBS and 2% sterile human urine at 27 °C (Figure 1A). Growth analysis revealed faster growth of the reference strain (COLPROT048) in Schneider’s medium compared to LIT, and a slight increase in cell number for *C. fasciculata* COLPROT606 in Schneider’s medium. *L. braziliensis* failed to grow in LIT medium, demanding the use of Schneider’s medium for subsequent experiments. Additionally, *C. fasciculata* COLPROT606 survived and replicated at 32 °C, although in fewer numbers than at 27 °C (Figure 1B). However, the parasites were not capable of surviving at 34 °C, resulting in an 80% cell death within 24 h (Figure 1C). Similar results were observed using *C. fasciculata* COLPROT048. Additionally, 32 °C is the temperature used to induce *Leishmania* amastigogenesis, and so it may mimic the temperature within mammalian host cells.

**Figure 2:**
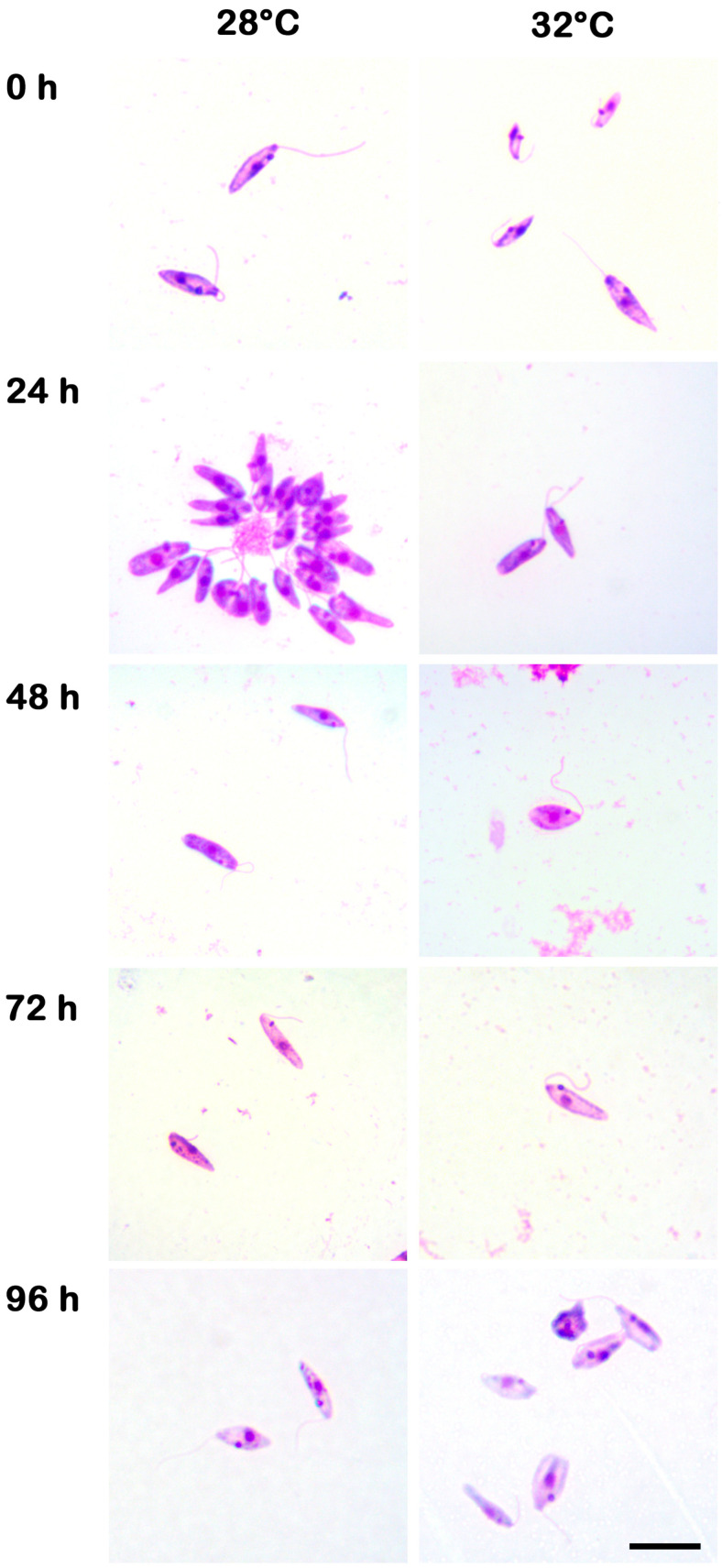
Morphology of *C. fasciculata* isolated from a patient (COLPROT606). Illustrative panel of both growth kinetics’ morphology (27 °C and 32 °C) of the COLPROT606 strain stained with Giemsa. Parasites were cultured for 5 days with an initial concentration of 1 × 10^6^ parasites/mL in Schneider’s medium supplemented with 20% FBS and 2% sterile human male urine. Bar: 10 µm.

Morphological parameters at different temperatures of the growth kinetics were assessed using Giemsa staining prepared every 24 h (Figure 2). Cells with subtle morphological differences were observed at 32 °C, whereas at 27 °C they maintained a less altered morphology with rosette formation, which indicates high cellular growth and adaptation. To assess *C. fasciculata* survival at elevated temperatures, mitochondrial membrane potential (ΔΨm) was measured, showing that both strains maintained mitochondrial function more effectively than *L. braziliensis*, indicating moderate thermotolerance (Table S1).

### 3.2. Cell Growth Evaluation in Leishmania-Crithidia Co-Cultures

A common approach to isolate parasites from clinical lesions requires the inoculation of the biopsy material in culture for taxonomic identification following cell growth. However, this methodology raises the concern of whether one parasite might overcome the other in the culture. To explore this question, we simulated an in vitro mixture under the following conditions: *L. braziliensis* alone (control), *C. fasciculata* COLPROT606 alone (control), a 1:1 mixture of *L. braziliensis* and *C. fasciculata*, and a 2:1 mixture of the two parasites The cultures were subcultured every 3 days, and after 5 passages the cells had their RNA extracted for quantitative RT-qPCR analysis.

**Figure 3:**
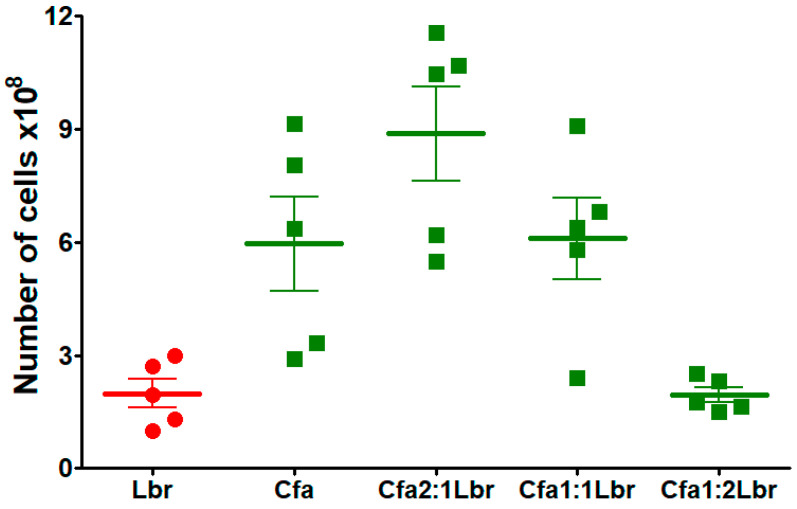
RT-qPCR analysis of cell growth in different ratio mixed cultures of *L. braziliensis*-*C. fasciculata* COLPROT606. *L. braziliensis* (red circles) and *C. fasciculata* COLPROT606 (green squares) at 1 × 10^6^ or 2 × 10^6^ parasites per 5 mL, were cultured in Schneider medium with 20% inactivated FBS at 27 °C in the following conditions: *L. braziliensis* alone (Lbr), *C. fasciculata* COLPROT606 alone (Cfa), and mixed cultures at ratios of 1:1, 2:1 and 1:2, amounting to 2 × 10^6^ parasites. Cultures were passed into fresh medium until the fifth passage for RNA extraction and RT-qPCR analysis with SSU rRNA primers and probes.

To quantify the number of each trypanosomatid in the co-infection’s assays, a sensitive and reproducible qPCR was developed. We standardized parasite load quantification by RT-qPCR employing the small subunit of the ribosomal RNA (SSU rRNA) primers and probes, which provides a higher specificity and sensibility to quantify each trypanosomatid without unspecific amplification (Figure S1). In the mixed cultures, the results demonstrated that *C. fasciculata* successfully outgrew *L. braziliensis* in all mixed infection conditions (Figure 3). The absolute quantification did not reveal the presence of *L. braziliensis* in any co-infection condition.

### 3.3. Susceptibility to Amphotericin B

**Figure 4:**
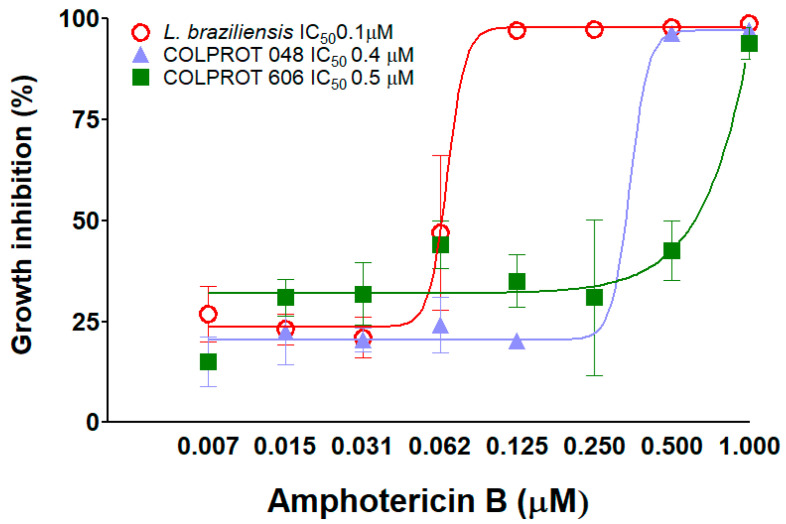
Dose–response curve of *L. braziliensis* (red circles), *C. fasciculata* COLPROT048 (purple triangles) and COLPROT606 (green squares) to Amphotericin B. 4 × 10^6^ parasites/mL were treated with a serial dilution of Amphotericin B starting from 1 µM for 72 h. After the incubation period, cell viability was assessed by fluorometry by adding 50 µM of resazurin (Alamar Blue^®^) per well. The reads were performed using a Spectra Max GEMINI XPS (Molecular Devices, Silicon Valley, San Jose, CA, USA) at an excitation of 560 nm and emission at 590 nm. The IC_50_ value at 72 h was determined using GraphPad Prism software v.8. Statistical test: Two-way ANOVA. *p* < 0.05.

Due to the increasing presence of non-pathogenic species in vertebrate hosts, which may exacerbate symptoms or promote resistance to leishmaniasis treatment [5,6], we evaluated *C. fasciculata* susceptibility to amphotericin B, a drug commonly used for leishmaniasis treatment. *L. braziliensis* showed an IC_50_/72 h of 0.1 ± 0.02 μM, while the values were 0.4 ± 0.02 and μM for and 0.5 ± 0.01 μM for COLPROT048 and COLPROT606, respectively (Figure 4).

### 3.4. In Vitro Coinfection of C. fasciculata and L. braziliensis

**Figure 5:**
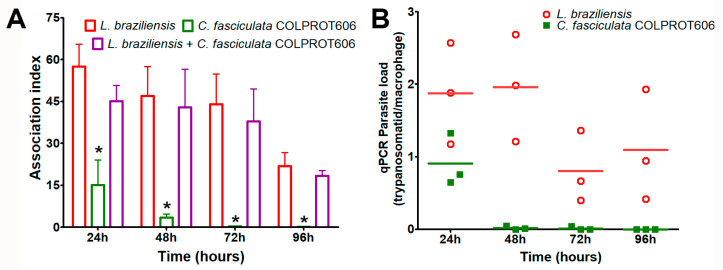
In vitro infection of BALB/c peritoneal macrophages with *L. braziliensis*, *C. fasciculata* COLPROT606, and coinfection with both parasites (1:1). (**A**) Macrophages were infected or co-infected in a ratio of 1:5 macrophages/parasites for 2 h and the number of infected cells and amastigotes were determined by light microscope counting and calculated using the formula: % number of infected macrophages × number of amastigotes/total number of macrophages. The values correspond to the association index at 24-, 48-, 72-, and 96 h post-infection. (**B**) Absolute quantification by RT-qPCR of the macrophage coinfection by *L. braziliensis* (circles) and *C. fasciculata* COLPROT606 (squares) at 24-, 48-, 72-, and 96 h post-infection. The quantification was performed employing the SSU rRNA primers and probes for each parasite, and tubulin beta (Mm.PT.39a.22214835, IDT Inc.) for normalization with mice cDNA. Statistical test performed: two-way ANOVA. (*) *p* < 0.001.

Macrophages are key immune cells controlling *Leishmania* infections, and the failure to control parasite replication within these cells is critical for disease progression. Here, we analyzed the ability of *C. fasciculata* parasites to infect macrophages, and maintain the infection for 96 h, by optical microscopy. As expected, *L. braziliensis* was internalized by macrophages and maintained infection for 4 days (Figure 5). In contrast, *C. fasciculata* strains (COLPROT048 and COLPROT606) showed a significantly lower number of parasites inside macrophages at 24 h post infection. This number persisted low at 48 and 72 h, decreasing even more at 96 h (Figure 5A). No statistical difference was observed between *C. fasciculata* COLPROT048 and COLPROT606 strains.

Considering that *L. braziliensis* might be able to modulate the immune response to support the survival of *C. fasciculata*, in vitro coinfection of murine macrophages was also evaluated by RT-qPCR. As observed previously, *L. braziliensis* alone was successfully internalized and maintained the macrophage for 96 h, but *C. fasciculata* COLPROT606 failed to persist alone in macrophages over the same period. In coinfected murine macrophages, 5 parasites of each species were added per macrophage, keeping the total number equal to that of the single-species control. *L. braziliensis* RNA was detected in high number at the 96 h of infection, but *C. fasciculata* was only detected at 24 h pos-infection (Figure 5B). These findings suggest that *L. braziliensis* does not enhance the survival of *C. fasciculata* within macrophages or exacerbate infection, but rather eliminated *C. fasciculata* from the vertebrate host cells.

### 3.5. In Vivo Experimental Infections

**Figure 6:**
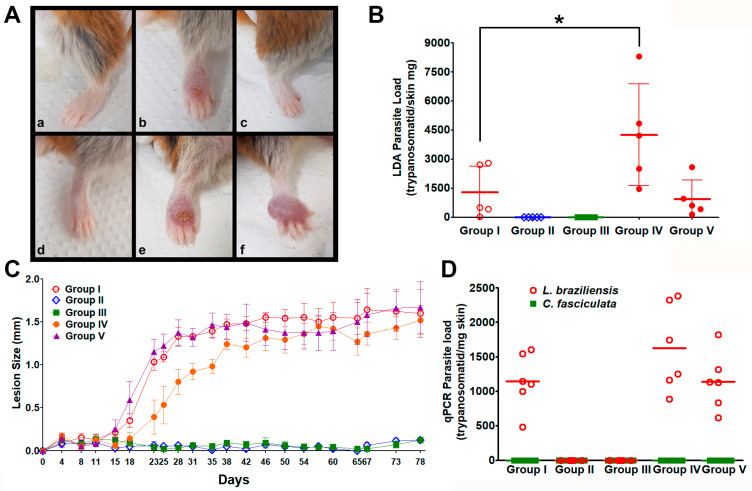
Experimental infection of golden hamsters’ right hind paw with *L. braziliensis* and *C. fasciculata***.** (**A**,**C**) Course of golden hamsters’ infection with *L. braziliensis* and *C. fasciculata* parasites. Non-infected hamster paw was illustrated as image (**a**). The right hind paw was infected with 2.0 × 10^6^ *L. braziliensis* alone Group I, red circle, image (**b**), *C. fasciculata* COLPROT048 alone Group II, blue diamond, image (**c**), *C. fasciculata* COLPROT606 alone Group III, green square, image (**d**), *L. braziliensis* in co-infection with COLPROT048 Group IV, orange circle, image (**e**) and with COLPROT606 Group V, purple triangle, image (**f**). Lesion size of the infected paws was monitored weekly using a dial caliper for 78 days post-infection. (**B**) Parasite burden at the end of the experiment. The infected paws were removed, macerated and analyzed by limiting dilution (LDA) in a microplate. No culture growth was observed in the wells from hamsters infected only with *C. fasciculata* strains COLPROT048 and COLPROT606 (**D**) Absolute quantification of parasitic load by RT-qPCR using SSU rRNA primers and probes specific for each parasite, with tubulin beta (Mm.PT.39a.22214835, IDT Inc.) as normalization for hamster cDNA. Statistical analysis: two-way ANOVA * *p* < 0.05.

The infectivity of *C. fasciculata* was evaluated in both BALB/c mice and golden hamsters, two established experimental models for *Leishmania* spp. infection. The golden hamsters are the most susceptible in vivo experimental model for *L. braziliensis* infection, capable of developing large and self-resolving lesions [23], Therefore, female hamsters were infected in the dorsum of their right hind paw with 1.0 × 10^6^ parasites according to the following protocol: Group I—*L. braziliensis* alone, Group II—*C. fasciculata* COLPROT048, Group III—*C. fasciculata* COLPROT606, Group IV—*L. braziliensis* co-infected with *C. fasciculata* COLPROT048, and Group V—*L. braziliensis* co-infected with *C. fasciculata* COLPROT606 (Figure 6).

Our results reported apparent lesions in control Group I, and in the co-infections of *L. braziliensis* with *C. fasciculata* COLPROT048 and COLPROT606, Groups IV and V, including the typical ulceration of this lesion in this experimental model (Figure 6A,C). However, no apparent lesions were observed on the paws of the hamsters infected only with *C. fasciculata*, Groups II and III. Moreover, the LDA parasite burden analysis did not report any parasite growth of in *C. fasciculata* strains infected alone, Groups II and III (Figure 6B). In the systems in which it was possible to observe a LDA positive parasite growth, Groups I, IV and V, the paw samples had a higher burden compared to popliteal lymph node.

In order to improve our co-infection analysis, we performed a qPCR quantification of the removed paws to detect *L. braziliensis* and *C. fasciculata* RNA, which could indicate the presence of live parasites at the end of the experiment. No *C. fasciculata* RNA was detected by RT-qPCR in the co-infection’s groups after 78 days of infection, or *Crithidia* alone (Figure 6D). However, *L. braziliensis* was detected in all the three groups where this parasite was used. A mean of 1145 parasites/mg skin equivalents were observed in Group I, 1625 parasites/mg in the co-infected Group IV, and 1141 parasites/mg in the co-infected Group V (Figure 6D). The changes in the qPCR parasite load were not statistically significant, suggesting that the co-infected were not capable of improving the hamster infection. The absence of *C. fasciculata* RNA in Groups II and III leads us to assume that this parasite is not able to maintain itself in hamsters.

**Figure 7:**
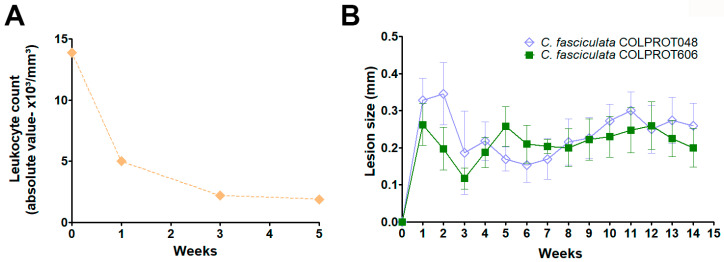
Experimental infection of immunosuppressed BALB/c cyclophosphamide-treated mice. (**A**) Uninfected BALB/c mice were treated intraperitoneally with cyclophosphamide at a dose of 150 mg/kg (3 mg/animal) and had their leukocytes counted weekly. (**B**) Cyclophosphamide-treated BALB/c females were infected on the left posterior paw with 1 × 10^7^ *C. fasciculata* COLPROT048 (blue diamonds) and *C. fasciculata* COLPROT606 (green squares). The size of the infected paws was monitored weekly using a dial caliper.

In the BALB/c mouse model, infection with *L. braziliensis* and *C. fasciculata* COLPROT048 and COLPROT606 induced significant inflammation, as measured by increased ear thickness (Figure S2A). However, in hamsters *C. fasciculata* COLPROT048 and COLPROT606 alone failed to induce any visible signs of infection or growth, indicating that *C. fasciculata* are also not capable of surviving or establishing an infection in BALB/c mice (Figure S2B). In addition, no *C. fasciculata* growth or RNA were observed. Notwithstanding, we decided to investigate the persistence of *C. fasciculata* in immunosuppressed BALB/c mice treated weekly with cyclophosphamide (3 mg/animal, intraperitoneally). The animals were infected in the left hind paw with *C. fasciculata* COLPROT048 and with the clinical isolate *C. fasciculata* COLPROT606. A control group received the same treatment without infection. Immunosuppression efficacy was confirmed by a decrease in leukocyte count from 13.9 to 1.9 over 5 weeks (Figure 7A). The infection was monitored by measuring paw thickness, and after 14 weeks, both the paws and draining popliteal lymph nodes were collected for LDA parasite burden analysis. An initial increase in paw thickness was observed, followed by a stabilization in the size of the lesion (Figure 7B), but no parasite growth was detected in cultures from the paws or lymph nodes of cyclophosphamide-treated mice. These results suggest that *C. fasciculata* is unable to persist in BALB/c mice, even under immunosuppression.

### 3.6. Co-Infection of Sandflies with L. braziliensis and C. fasciculata COLPROT606

**Figure 8:**
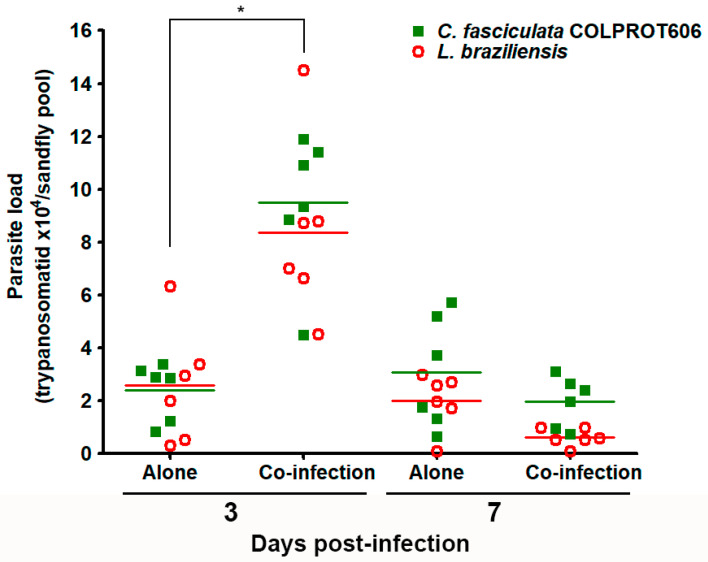
In vivo experimental infection and co-infection of *Lu. longipalpis* with *L. braziliensis* and *C. fasciculata* COLPROT606. The sandflies were infected with 5.0 × 10^6^ parasites/mL of *L. braziliensis* (red circles), *C. fasciculata* COLPROT606 (green squares) and a 1:1 mixture of both parasites. The RNA from infected sandflies were extracted from their guts on days 3- and 7-post-infection to quantify the number of *L. braziliensis* and *C. fasciculata* COLPROT606 per sandfly pool of 10 insects by RT-qPCR employing SSU rRNA primers and SYBR Green. The horizontal straight lines indicate the median value of *L. braziliensis* (red line) and *C. fasciculata* (green line). Statistical test performed: one-way ANOVA, * *p* < 0.05.

To assess the transmissibility of *C. fasciculata* in an invertebrate host, we tested its ability to colonize the *L. longipalpis* sandfly, a known permissive vector of *Leishmania* spp. [24]. Female sandflies (100 per group) were fed with blood containing: (i) *L. braziliensis*, (ii) *C. fasciculata* (COLPROT606), or (iii) both parasites (1:1). A pool of 10 insects was collected on days 3 and 7 post-infection and analyzed by RT-qPCR. Results showed that *C. fasciculata* and *L. braziliensis* were present at the two time points, both alone and in coinfection, (Figure 8). These findings suggest that *C. fasciculata* can colonize *L. longipalpis*, offering insights into the transmission dynamics of these trypanosomatids in vertebrate hosts. Moreover, the co-infection significantly increased the number of both parasites on the third day, rising from 2.4 × 10^4^ to 9.5 × 10^4^ *C. fasciculata*/sandfly and 2.5 × 10^4^ to 8.3 × 10^4^ *L. braziliensis*/sandfly. On day 7, the number of both parasites decreased and no statistical difference was observed both alone and in the mixed infection (Figure 8).

## 4. Discussion

Recent reports of human infections by monoxenous trypanosomatids, previously assumed to be insect-exclusive, are raising concerns about their pathogenic potential in vertebrates, including humans. *Leptomonas seymouri* has been frequently identified in co-infections with *Leishmania donovani* in the Indian subcontinent, often associated with more severe clinical outcomes and possible drug resistance [15,16]. Similarly, *Crithidia* spp. have been reported in both humans and animals, with cases documented in Brazil and Iran [4,14]. In humans, monoxenous infections are primarily described in immunocompromised individuals co-infected with HIV and *L. major* or *L. infantum*, but have also been detected in immunocompetent hosts as the sole pathogen [4,5,6,7,8,9,10,11,12,13,14]. Additionally, species of *Herpetomonas* and *Blechomonas* (formerly classified as *Leptomonas*) have been sporadically detected in humans [7]. These emerging reports suggest an expanding host range and increased adaptability of monoxenous trypanosomatids, reinforcing the need for intensified surveillance and continued investigation into their pathogenic mechanisms. Therefore, in this study, we further investigated the biological and pathogenic characteristics of a *C. fasciculata* isolate obtained from a skin lesion of a patient from Cusco, Peru [4,17], to better understand its behavior under experimental conditions and its potential role in human infections.

Our findings demonstrate that *C. fasciculata* was capable of surviving and proliferating in two media specifically designed for monoxenous trypanosomatids, i.e., LIT and Schneider’s medium, the last typically used for *Leishmania* species. Furthermore, the parasite’s limited growth at 32 °C, a temperature usually employed for *Leishmania* axenic amastigogenesis [25], indicates a certain degree of thermotolerance, although the reduction in *C. fasciculata* ΔΨm at 32 °C after 96 h suggests the loss of parasites’ viability in this adverse condition. The capability to withstand a higher temperature supports previous observations of the parasite’s ability to grow in environments with low nutrient availability and inconstant temperatures, such as the conditions in the regions where *C. fasciculata* has been reported causing mammal infections [4]. Interestingly, in co-culture experiments, *C. fasciculata* outgrew *L. braziliensis*, highlighting its faster growth rate under laboratory conditions. However, in contrast to prior studies with *Crithidia* coinfection, we observed that *C. fasciculata* COLPROT606 failed to survive at higher temperatures, such as 34 °C, suggesting that environmental temperature may limit some isolates to thrive in warmer conditions [14,26].

Following these findings, it is important to consider the “environment-biased selection hypothesis”, which suggests that in co-infection scenarios, laboratory culture conditions may favor the faster-growing monoxenous trypanosomatids, such as *C. fasciculata*, potentially overshadowing the growth of *Leishmania* species [27]. In our co-culture experiments, *C. fasciculata* consistently outgrew *L. braziliensis*, making it unfeasible to detect *Leishmania* parasites after extended incubation periods. This result supports the hypothesis mentioned, indicating that *C. fasciculata* outgrew the pathogenic species in laboratory conditions due to the monoxenous faster growth, which could lead to potential diagnostic misinterpretations in co-infections. On the other hand, in the vertebrate model, *Leishmania* outcompetes the monoxenous trypanosomatids, which may be eliminated by host immune responses, or even by the lack of other mechanisms to establishes infection [27].

Unlike pathogenic *Leishmania* species, *C. fasciculata* did not survive or proliferate within murine peritoneal macrophages despite an initial infection, indicating the absence of immune evasion mechanisms commonly associated with *Leishmania* infections [28,29]. However, *Crithidia* sp. CLA-KP1, isolated from the biting midge *Culicoides peregrinus*, was cleared from murine peritoneal exudate macrophages (PEMs) by 48 h [26]; whereas, the Brazilian clinical isolate *Crithidia* sp. LVH60 infected THP-1 cells for up to 72 h [11]. Another *C. fasciculata* isolated in Iran infected both J774 and THP-1 cells, yet the persistence was not specified [6]. The results presented here are aligned with findings on *Leptomonas seymouri*, which also failed to persist in mammalian macrophages during co-infection with *L. donovani* [15]. Together, these findings suggest that *C. fasciculata* isolated from Peru could lack the virulence factors required to cause disease in vertebrate hosts. Furthermore, its inability to establish infection in murine or golden hamster models, even under immunosuppressed conditions, further supports the conclusion that *C. fasciculata* could be non-pathogenic under the experimental conditions assayed here. Nevertheless, we emphasize that only a single mammalian isolate was tested and that experiments were conducted with a limited number of replicates, which restricts the generalization of our conclusions. Further in vivo and in vitro studies with additional isolates are necessary to clarify whether our findings are consistent across different strains and host contexts.

Another key aspect of our study was assessing *C. fasciculata* resistance to Amphotericin B, a drug commonly used for the treatment of leishmaniasis [30]. Our results showed that *C. fasciculata* parasites isolated from a human patient exhibited greater resistance to Amphotericin B compared to both *L. braziliensis* and the reference *C. fasciculata* strain. This result suggests that *C. fasciculata* may harbor intrinsic mechanisms of drug resistance, as supported by a previous study comparing growth inhibition between *C. fasciculata* and *Leptomonas*, where *C. fasciculata* required 3 to 6 times higher concentrations of phenanthridines and diamidines to achieve 50% inhibition compared to *Leptomonas* [31]. Although this result is intriguing, it should be interpreted with caution, since resistance was assessed only for Amphotericin B and exclusively in vitro. Additional experiments using different drugs and clinical isolates are required to evaluate the potential clinical implications of this phenomenon, especially in co-infection scenarios where resistance traits could be transferred or masked.

In the sandfly insect host, *C. fasciculata* was detected in all samples after artificial blood infection, indicating its ability to establish in the vector. A meta-analysis has shown that insects exhibit a higher prevalence of monoxenous trypanosomatids, likely due to a trade-off favoring dissemination over complex host adaptation [32]. This increased prevalence could favor contact with vertebrate hosts, presenting a challenge which should be overcome by the monoxenous parasite. In the presence of a dixenous parasite the survival of the monoxenous counterpart could be favored by a synergistic interaction between both trypanosomatids. In this context, studies reporting sandflies naturally infected with monoxenous trypanosomatids suggest that human infection with *C. fasciculata* could share similar transmission dynamics with pathogenic species [33,34,35,36,37]. Notwithstanding, the passage of the parasite through the vector could select for more virulent populations [37], and factors such as immunomodulatory molecules presented in sandfly saliva may also influence the success of infection [38]. Additional research should aim to evaluate these natural transmission conditions more closely to better understand how the vector influences the pathogenic potential of *C. fasciculata*, and to clarify the interaction dynamics of trypanosomatids in their natural environments.

Taken together, these findings underscore the need for improved diagnostic accuracy. Clinical isolates of *C. fasciculata* can be easily mistaken for *Leishmania* due to morphological similarity and the limitations of conventional tests, such as direct smears or immunological assays, especially in endemic areas [7,11]. Therefore, the use of molecular methods, including PCR and sequencing, is essential, as they have proven more effective in distinguishing *C. fasciculata* from pathogenic *Leishmania*, as demonstrated by recent qPCR-based studies in clinical isolates [7,11].

## 5. Conclusions

Over the last few years, an increased number of reports of monoxenous trypanosomatids in vertebrate hosts have emerged. However, the mechanisms involved in the pathogenesis and transmission of such parasites in co-infection with pathogenic trypanosomatids remain poorly understood. Our findings suggest that the *Crithidia fasciculata* COLPROT606 isolate from an atypical human infection can grow in vitro under moderate temperatures, colonize permissive sandfly vectors, outcompete *L. braziliensis* in co-culture, and exhibits partial resistance to Amphotericin B. However, it fails to persist or proliferate within mammalian macrophages and does not establish infection in immunocompetent or immunosuppressed BALB/c mice or golden hamsters, nor does co-infection with *L. braziliensis* enhance its survival. These results indicate that *C. fasciculata* COLPROT606 lacks the virulence and adaptive mechanisms required for sustained infection in vertebrate hosts, underlining the importance of molecular screening of atypical lesions and further investigation into its transmission and ecological interactions.

## Figures and Tables

**Table 1:** Primers and probes sequences used for RT-qPCR quantification of *C. fasciculata*, *L. braziliensis*, and mouse β-tubulin (Mm.PT.39a).

Target	Primer/Probe	Sequence (5′-3′)
*C. fasciculata* SSU rRNA	Forward	CCGTGCCCTCAAGAACAT
	Reverse	GGGATGTTCACACCGTACAA
	Probe	FAM-TGCACAAGAAGAAGCAGGAGCAGA-3IABkFQ
*L. braziliensis* SSU rRNA	Forward	TGACGAACCCACACAACAA
	Reverse	GGTCGCGAATTATCTCCCAATA
	Probe	HEX-ACCGAACGAAAGCTGAACCACACT-3IABkFQ
Mouse β-tubulin (Mm.PT.39a.22214835, IDT Inc.)	Forward	GGGTGGAACTGTGTTACGTAG
	Reverse	TGGTCTTTCTGGTGCTTGTC
	Probe	Cy5-CCGGAGAATGGGAAGCCGAACATAc-3IAbRQSp

## Data Availability

The original contributions presented in this study are included in the article/Supplementary Materials. Further inquiries can be directed to the corresponding author.

## Supplementary Materials

The following supporting information can be downloaded at https://www.mdpi.com/article/10.3390/microorganisms13102335/s1, Figure S1: Standardization of qPCR assays for parasite load quantitation from culture mixtures, in vitro peritoneal macrophage infection and in vivo experiments with *L. braziliensis* and *C. fasciculata* COLPROT 606; Figure S2: Experimental infection in the right ear of BALB/c mice. Table S1: Δψm analysis of *C. fasciculata* COLPROT048, *C. fasciculata* COLPROT606 and *L. braziliensis* at 27 °C and 32 °C.

## References

[1] Hamida A., Ilyas D., Zahra M., Shehnaz G. (2019). Leishmaniasis: A Neglected Tropical Disease. Glob. Immunol. Infect. Dis. Rev..

[2] Karmakar S., Volpedo G., Zhang W.-W., Lypaczewski P., Ismail N., Oliveira F., Oristian J., Meneses C., Gannavaram S., Kamhawi S. (2022). Centrin-deficient *Leishmania mexicana* confers protection against Old World visceral leishmaniasis. NPJ Vaccines.

[3] Maslov D.A., Votýpka J., Yurchenko V., Lukeš J. (2013). Diversity and phylogeny of insect trypanosomatids: All that is hidden shall be revealed. Trends Parasitol..

[4] Toledo P.D. (2007). Comportamiento In Vitro de um Kinetoplastido Trypanosomatidae Aislado de uma Lesion Cutanea. Licenciate Thesis.

[5] Maruyama S.R., de Santana A.K., Takamiya N.T., Takahashi T.Y., Rogerio L.A., Oliveira C.A., Milanezi C.M., Trombela V.A., Cruz A.K., Jesus A.R. (2019). Non-Leishmania parasite in fatal visceral leishmaniasis-like disease, Brazil. Emerg. Infect. Dis..

[6] Ghobakhloo N., Motazedian M.H., Naderi S., Ebrahimi S. (2019). Isolation of *Crithidia* spp. from lesions of immunocompetent patients with suspected cutaneous leishmaniasis in Iran. Trop. Med. Int. Health.

[7] Boucinha C., Andrade-Neto V.V., Ennes-Vidal V., Branquinha M.H., Santos A.L.S., Torres-Santos E.C. (2022). A stroll through the history of monoxenous trypanosomatids infection in vertebrate hosts. Front. Cell. Infect. Microbiol..

[8] Fakhar M., Derakhshani-Nia M., Gohardehi S., Karamian M., Hezarjaribi H.Z., Mohebali M., Akhoundi B., Sharbatkhori M. (2022). Domestic dogs carriers of *Leishmania infantum*, *Leishmania tropica*, and *Crithidia fasciculata* as potential reservoirs for human visceral leishmaniasis in northeastern Iran. Vet. Med. Sci..

[9] Rodrigues E.S., Santos G.Q., da Silva M.V., Barros J.H.S., Bernardo A.R., Diniz R.L., Rubim N.M., Roque A.L.R., Jansen A.M., Silva E.D. (2022). Chagas immunochromatographic rapid test in the serological diagnosis of *Trypanosoma cruzi* infection in wild and domestic canids. Front. Cell. Infect. Microbiol..

[10] Rogerio L.A., Takahashi T.Y., Cardoso L., Takamiya N.T., de Melo E.V., de Jesus A.R., de Oliveira F.A., Forrester S., Jeffares D.C., da Silva J.S. (2023). Co-infection of *Leishmania infantum* and a *Crithidia*-related species in a case of refractory relapsed visceral leishmaniasis with non-ulcerated cutaneous manifestation in Brazil. Int. J. Infect. Dis..

[11] Takamiya N.T., Rogerio L.A., Torres C., Leonel J.A.F., Vioti G., Oliveira T.M.F.d.S., Valeriano K.C., Porcino G.N., Santos I.K.F.d.M., Costa C.H.N. (2023). Parasite detection in visceral leishmaniasis samples by dye-based qPCR using new gene targets of *Leishmania infantum* and *Crithidia*. Trop. Med. Infect. Dis..

[12] Fadhil S.A., Ali M.J. (2024). Molecular identification of *Crithidia* sp. from naturally infected dogs. Iraqi J. Vet. Sci..

[13] Khademi A., Mohammadi Z., Tohidi F. (2024). Investigating *Crithidia* spp. in ulcer smear of patients suspected of leishmaniasis in Aq-Qala, Golestan province, Northern Iran, 2019–2020. Jorjani Biomed. J..

[14] Boucinha C., Caetano A.R., Santos H.L., Helaers R., Vikkula M., Branquinha M.H., dos Santos A.L.S., Grellier P., Morelli K.A. (2020). Analysing ambiguities in trypanosomatids taxonomy by barcoding. Mem. Inst. Oswaldo Cruz.

[15] Kraeva N., Butenko A., Hlaváčová J., Kostygov A., Myškova J., Grybchuk D., Leštinová T., Votýpka J., Volf P., Opperdoes F. (2015). *Leptomonas seymouri*: Adaptations to the dixenous life cycle analyzed by genome sequencing, transcriptome profiling and co-infection with *Leishmania donovani*. PLoS Pathog..

[16] Sukla S., Nath H., Kamran M., Ejazi S.A., Ali N., Das P., Ravichandiran V., Roy S., Biswas S. (2022). Detection of *Leptomonas seymouri* in an RNA-like virus in serum samples of visceral leishmaniasis patients and its possible role in disease pathogenesis. Sci. Rep..

[17] Ennes-Vidal V., Vitório B.S., Menna-Barreto R.F.S., Pitaluga A.N., Gonçalves-da-Silva S.A., Branquinha M.H., Santos A.L.S. (2019). Calpains of *Leishmania braziliensis*: Genome analysis, differential expression, and functional analysis. Mem. Inst. Oswaldo Cruz.

[18] Bombaça A.C.S., Gandara A.C.P., Ennes-Vidal V., Bottino-Rojas V., Dias F.A., Farnesi L.C., Sorgine M.H., Bahia A.C., Bruno R.V., Menna-Barreto R.F.S. (2021). *Aedes aegypti* infection with trypanosomatid *Strigomonas culicis* alters midgut redox metabolism and reduces mosquito reproductive fitness. Front. Cell. Infect. Microbiol..

[19] Santa-Rita R.M., Henriques-Pons A., Barbosa H.S., De Castro S.L. (2004). Effect of the lysophospholipid analogues edelfosine, ilmofosine and miltefosine against *Leishmania amazonensis*. J. Antimicrob. Chemother..

[20] Andrade-Neto V.V., Cunha-Júnior E.F., Canto-Cavalheiro M.M.D., Atella G.C., Fernandes T.d.A., Costa P.R.R., Torres-Santos E.C. (2016). Antileishmanial activity of Ezetimibe: Inhibition of sterol biosynthesis, In Vitro synergy with azoles, and efficacy in experimental cutaneous leishmaniasis. Antimicrob. Agents Chemother..

[21] Inacio J.D.F., Gervazoni L., Canto-Cavalheiro M.M., Almeida-Amaral E.E. (2014). The effect of (-)-epigallocatechin 3-O In Vitro and In Vivo in *Leishmania braziliensis*: Involvement of reactive oxygen species as a mechanism of action. PLoS Negl. Trop. Dis..

[22] Meireles A.C.A., Amoretty P.R., Souza N.A., Kyriacou C.P., Peixoto A.A. (2006). Rhythmic expression of the cycle gene in a hematophagous insect vector. BMC Mol. Biol..

[23] Gomes-Silva A., Valverde J.G., Ribeiro-Romão R.P., Placido-Pereira R.M., Da-Cruz A.M. (2013). Golden hamster (*Mesocricetus auratus*) as an experimental model for *Leishmania (Viannia) braziliensis* infection. Parasitology.

[24] Volf P., Myskova J. (2007). Sand flies and *Leishmania*: Specific versus permissive vectors. Trends Parasitol..

[25] de Melo L.V., Vasconcelos dos Santos T., Ramos P.K., Lima L.V., Campos M.B., Silveira F.T. (2024). Antigenic reactivity of *Leishmania (Viannia) lainsoni* axenic amastigote proved to be a suitable alternative for optimizing Montenegro skin test. Parasites Vectors.

[26] Kaewmee S., Mano C., Phanitchakun T., Ampol R., Yasanga T., Pattanawong U., Junkum A., Siriyasatien P., Bates P.A., Jariyapan N. (2023). Natural infection with *Leishmania (Mundinia) martiniquensis* supports *Culicoides peregrinus* (Diptera: Ceratopogonidae) as a potential vector of leishmaniasis and characterization of a *Crithidia* sp. isolated from the midges. Front. Microbiol..

[27] Domagalska M.A., Dujardin J. (2020). Non-*Leishmania* parasite in fatal visceral leishmaniasis-like disease, Brazil. Emerg. Infect. Dis..

[28] Barral-Netto M., Badaró R., Barrai A., Carvalho E.M. (1986). Imunologia da Leishmaniose Tegumentar. Rev. Soc. Bras. Med. Trop..

[29] Kaye P., Scott P. (2011). Leishmaniasis: Complexity at the host-pathogen interface. Nat. Rev. Microbiol..

[30] Alves L.L., Freire M.L., Troian I.L., Morais-Teixeira E., Cota G. (2024). Local amphotericin B therapy for cutaneous leishmaniasis: A systematic review. PLoS Negl. Trop. Dis..

[31] Bacchi C.J., Lambros C., Goldberg B., Hutner S.H., De Carvalho G.D.F. (1974). Susceptibility of an insect *Leptomonas* and *Crithidia fasciculata* to several established anti-trypanosomatid agents. Antimicrob. Agents Chemother..

[32] Al-Ghafli H., Barribeau S.M. (2023). Double trouble: Trypanosomatids with two hosts have lower infection prevalence than single host trypanosomatids. Evol. Med. Public Health.

[33] Ferreira T.d.S., Minuzzi-Souza T.T.C., de Andrade A.J., Coelho T.O., Rocha D.d.A., Obara M.T., Hecht M., Nitz N., Gurgel-Gonçalves R. (2015). Molecular detection of *Trypanosoma* sp. and *Blastocrithidia* sp. (Trypanosomatidae) in phlebotomine sand flies (Psychodidae) in the Federal District of Brazil. Rev. Soc. Bras. Med. Trop..

[34] Kalantari M., Motazedian M.H., Asgari Q., Soltani Z., Soltani A., Azizi K. (2018). Bionomics of phlebotomine sand flies species (Diptera: Psychodidae) and their natural infection with *Leishmania* and *Crithidia* in Fars Province, Southern Iran. J. Parasit. Dis..

[35] Tanure A., Rêgo F.D., Tonelli G.B., Campos A.M., Shimabukuro P.H.F., Gontijo C.M.F., Paz G.F., Andrade-Filho J.D. (2020). Diversity of phlebotomine sand flies and molecular detection of trypanosomatids in Brumadinho, Minas Gerais, Brazil. PLoS ONE.

[36] Songumpai N., Promrangsee C., Noopetch P., Siriyasatien P., Preativatanyou K. (2022). First evidence of co-circulation of emerging *Leishmania martiniquensis*, *Leishmania orientalis*, and *Crithidia* sp. in *Culicoides* biting midges (Diptera: Ceratopogonidae), the putative vectors for autochthonous transmission in Southern Thailand. Trop. Med. Infect. Dis..

[37] Ennes-Vidal V., Pitaluga A.N., Britto C.F.P.C., Branquinha M.H., dos Santos A.L.S., Menna-Barreto R.F.S., D’aVila-Levy C.M. (2020). Expression and cellular localisation of *Trypanosoma cruzi* calpains. Mem. Inst. Oswaldo Cruz.

[38] Fayaz S., Bahrami F., Parvizi P., Fard-Esfahani P., Ajdary S. (2022). An overview of the sand fly salivary proteins in vaccine development against leishmaniases. Iran J. Microbiol..

