# Supplementary material for: Crithidia fasciculata Shows Non-Pathogenic Behavior in Leishmania Co-Infection Related to Temperature Stress, In Vitro and In Vivo Infections, and Amphotericin B Susceptibility
Source: Microorganisms. 2025 Oct 10;13(10):2335. doi: 10.3390/microorganisms13102335 (PMC12566015; doi:10.3390/microorganisms13102335)
Supplement: Supplementary file 1 [file microorganisms-13-02335-s001.zip › microorganisms-3823438-supplementary.pdf]

Table S1. Δψm analysis of *C. fasciculata* COLPROT048, *C. fasciculata* COLPROT606 and *L. braziliensis* at 27°C and 32°C.

Trypanosomatid		48 hours		96 hours	
		**TMRE+ cells (%)**	**IV[a]**	**TMRE+ cells (%)**	**IV[a]**
***C. fasciculata* COLPROT048**	27°C	91.0±1.1[b]	0.00	84.1±1.7	0.00
	27°C +CCCP10 μM	20.1±1.8*	-0.58*	14.9±1.0*	-0.95*
	32°C	82.8±2.0	0.07	79.6±0.5*	-0.60*
***C. fasciculata* COLPROT606**	27°C	87.5±1.7	0.00	74.4±0.8	0.00
	27°C +CCCP10 μM	29.9±2.0*	-0.63*	26.8±1.9*	-0.81*
	32°C	66.1±6.6*	-0.62*	52.1±1.3*	-0.64*
L. braziliensis	27°C	86.0±0.7	0.00	73.8±2.6	0.00
	27°C +CCCP10 μM	3.3 ±4.1*	-0.57*	10.0±2.1*	-0.96*
	32°C	51.8±4.1*	-0.30*	13.2±0.9*	-0.90*

[a]IV = (ME – MC)/MC, where ME corresponds to the median of fluorescence for parasites in experimental conditions of 32°C, and MC corresponds to control parasites at 27°C. The fluorescence of CCCP was reduced from ME and MC of each trypanosomatid.

[b]Mean ± standard deviation of 3 independent experiments

Asterisks indicate significant differences to the control group at 27°C ($p \leq 0.05$).

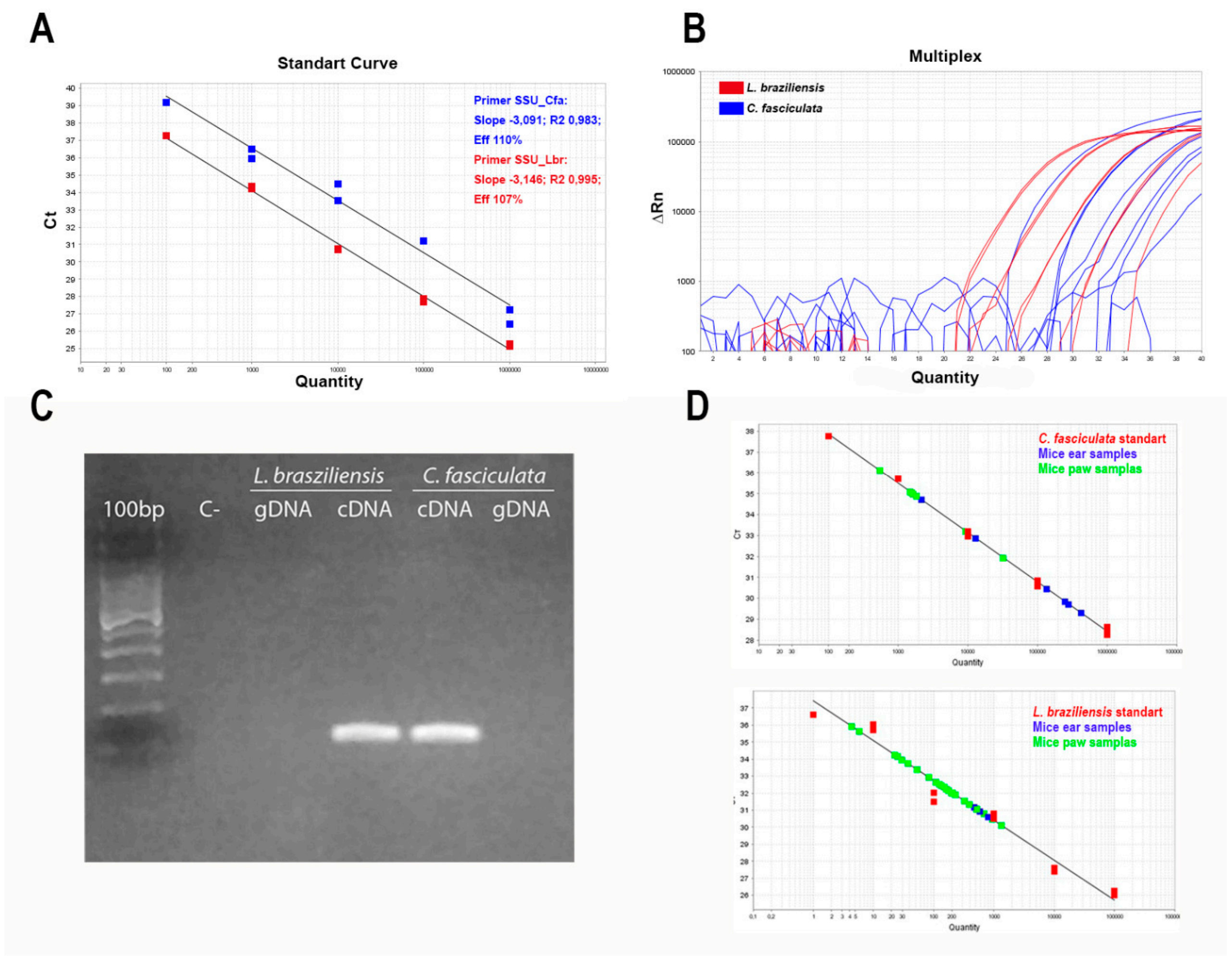

Figure S1. Standardization of qPCR assays for parasite burden quantification in culture mixtures, in vitro peritoneal macrophage infections, and in vivo experiments with *L. braziliensis* and *C. fasciculata* COLPROT606. (**A**) Representative standard curves for SSU amplification of both parasites, showing dynamic range, PCR efficiency, and linearity (R^2) of the reaction. (**B**) Amplification plots displaying fluorescence signal intensity for *L. braziliensis* and *C. fasciculata* COLPROT606 SSU targets. (**C**) Conventional PCR of *L. braziliensis* and *C. fasciculata* cDNA and genomic DNA (gDNA) using *L. braziliensis* actin primers, previously described in [17]. A 100 bp molecular marker was run in parallel to confirm the expected amplicon size. (**D**) Standard curves obtained from tissues of mice artificially infected with *C. fasciculata* and *L. braziliensis*. All cDNA samples were diluted 10-fold prior to qPCR, and parasite burdens were normalized accordingly. The qPCR assays were performed with SSU primers and probes as described in the Materials and Methods section.

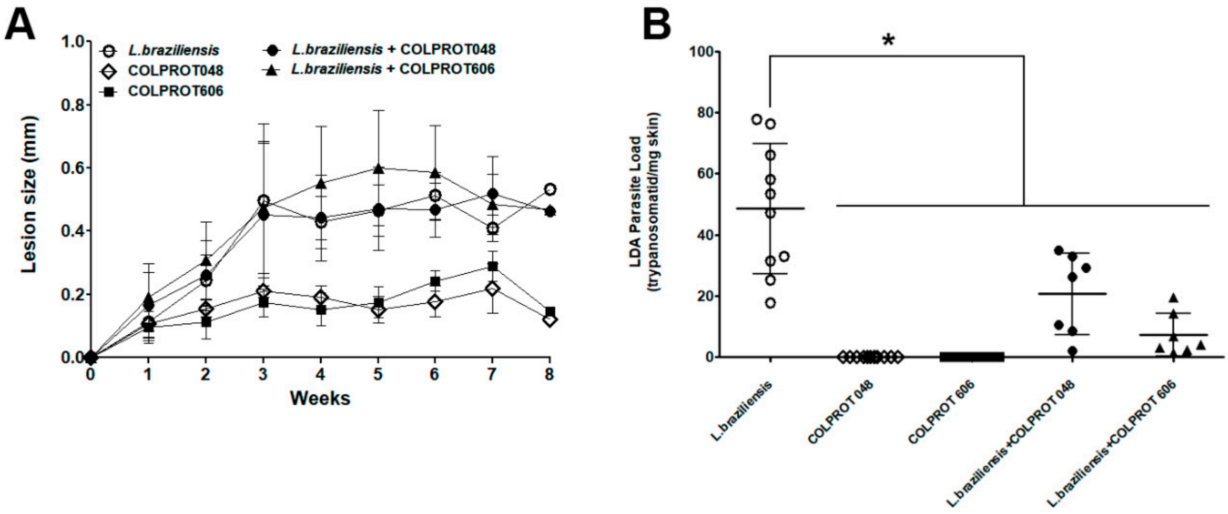

Figure S2. Experimental infection of the right ear in BALB/c mice. (**A**) Female BALB/c mice were infected in the right ear with 1×10^7 parasites from five groups: I) *L. braziliensis* alone (open circle); II) *C. fasciculata* COLPROT048 (open diamond); III) *C. fasciculata* COLPROT606 (closed square); IV) *L. braziliensis* + *C. fasciculata* COLPROT048 (closed circle); and V) *L. braziliensis* + *C. fasciculata* COLPROT606 (closed triangle). Increased ear thickness was observed in the *L. braziliensis* control group and in the coinfection groups IV and V. Ear thickness was measured weekly using a caliper. (**B**) At the final of the experiments, mice were euthanized and the infected ears were excised and homogenized for parasite burden analysis. Statistical analysis: one-way ANOVA; $P \leq 0.05$.
